# Using an Information Package to Reduce Patients’ Risk of Renal Damage: Protocol for a Randomized Feasibility Trial

**DOI:** 10.2196/29161

**Published:** 2021-04-30

**Authors:** Sharon Leitch, Alesha Smith, Jiaxu Zeng, Tim Stokes

**Affiliations:** 1 Department of General Practice and Rural Health Otago Medical School – Dunedin Campus University of Otago Dunedin New Zealand; 2 School of Pharmacy University of Otago Dunedin New Zealand; 3 Department of Preventive and Social Medicine Otago Medical School – Dunedin Campus University of Otago Dunedin New Zealand

**Keywords:** triple whammy, medication safety, patient education, general practice, NSAID, digital intervention, primary care, safety, protocol, feasibility, randomized controlled trial, risk, kidney, renal, information, acceptability

## Abstract

**Background:**

Non-steroidal anti-inflammatory drugs (NSAIDs) are a common cause of renal damage, especially when taken together with angiotensin-converting enzyme inhibitors (ACE-i) or angiotensin II receptor blockers (ARBs) plus a diuretic — a combination known as the “triple whammy.” New Zealand patients are at high risk of the “triple whammy” because they can easily purchase NSAIDs without a prescription and in nonpharmacy retail settings (eg, the supermarket), there is no legal requirement to include patient information sheets with medication, and direct-to-consumer drug advertising is permitted. A patient information package has been developed for those at greatest risk of the “triple whammy,” consisting of a printable PDF and an interactive online learning activity. This information package aims to inform patients about their elevated risk of harm from NSAIDS and discourage use of NSAIDs. A randomized control trial was planned to assess the effect of the information package.

**Objective:**

This study aims to pilot the trial procedures for recruiting patients and providing patient information online and to assess the acceptability of the patient information package.

**Methods:**

A two-armed randomized feasibility trial will be undertaken in Northland, New Zealand. We will recruit 50 patients who are at least 18 years old from those who have signed up to receive email alerts through their general practice. Patients eligible for this study have been prescribed an ACE-i or ARB plus a diuretic in the past 3 months. They will be randomly allocated to 2 study arms. The intervention arm will receive access to an information package plus usual care; the control arm will receive usual care alone. Online surveys will be used to assess NSAID knowledge and NSAID use at baseline and after 2 weeks for both arms. The intervention arm will also evaluate the information package in an additional survey based on Normalization Process Theory (NPT) concepts. We will report the number and proportion of participants who are eligible and consent to participate in the trial. Response and drop-out rates will be reported for each trial arm. The numbers of patients who interact with the education package will be reported together with the patient evaluation of it.

**Results:**

Funding has been obtained from the Health Research Council of New Zealand (HRC 18-031). The University of Otago Human Research Ethics Committee (H21/016) has approved this trial. Consultation has been undertaken with The Ngai Tahu research consultation committee. The trial commenced on April 12, 2021.

**Conclusions:**

This feasibility trial will test the study processes prior to commencing a randomized controlled trial and will determine the acceptability of the patient information package. We anticipate this work will provide useful information for other researchers attempting similar work.

**International Registered Report Identifier (IRRID):**

PRR1-10.2196/29161

## Introduction

Non-steroidal anti-inflammatory drugs (NSAIDs) are a common cause of renal damage [1]. The risk of renal injury dramatically increases when NSAIDs are taken together with angiotensin-converting enzyme inhibitors (ACE-i) or angiotensin II receptor blockers (ARBs) plus a diuretic [2] — this is known as the “triple whammy” combination. Despite this issue being well known and publicized heavily to prescribers in recent years, coprescribing of these medications is still common in New Zealand; 2017 data suggest more than 26,500 patients were prescribed these medications concurrently, with the majority of these patients aged over 65 years [3,4].

While prescribers should be well aware of the dangers of combining NSAIDs with other medications that alter renal perfusion, patients may not be [5-7]. The true extent of simultaneous use of these medications is unknown. Nonprescription use of NSAIDs may be high in New Zealand. This is due to the easy availability of NSAIDs for patients to purchase without a prescription and in nonpharmacy retail settings (eg, the supermarket), no legal requirement to include patient information sheets with medication, and direct-to-consumer drug advertising [8]. Given these issues, it is important to maximize the opportunities to advise patients about the potential risks of taking anti-inflammatories as part of the “triple whammy.”

Existing information for patients is generic and not generally considered fit-for-purpose [8]. Tailoring medication information for patients may improve the quality and usefulness of information, as well as improve patient knowledge [9,10]. However, examples of personalized medication information are rare [11]. Most patients consider doctors to be their main source of health information. Patients want to be able to share that information with their whanau (family) as well as health professionals [12,13], but doctors do not always provide patients with that information [14,15]. Doctors find communicating risk information difficult [16,17], and communicating potential risks of NSAIDs may be incomplete [18]. Time, low health literacy levels, and lack of patient interest have been suggested as additional barriers to communication [13].

Providing information directly to patients may help address some of those issues; however, information must be provided at an appropriate health literacy level. New Zealanders typically have low levels of health literacy — over half of all adults surveyed had skills “insufficient to cope with the health literacy demands they typically face” [19]. When the health literacy study results were broken down by ethnicity, 75%-80% of Maori (the indigenous people of Aotearoa, New Zealand) and 90% of Pasifika (people living in New Zealand who migrated from or have ancestry in the Pacific Islands) had low health literacy levels [19]. Health literacy has been identified as a cause of health disparities [20], and decision support tools can help address deficits in health literacy [21,22]. Self-efficacy, a patient’s belief in their ability to undertake and successfully complete a task, is another factor highly associated with optimal medication use [23,24].

Most medication information for patients is in leaflet form, with patient information resources becoming increasingly available online. Patients search for high-quality, reputable information, but find it hard to judge the quality of the information they find online [13]. A few studies have examined novel modalities of delivering health information to patients, but there is room for further research to evaluate the efficacy of alternative media modalities [25,26].

Conporto Health Event Detection & Mitigation (Conporto) is a software package that detects whether general practice patients are at risk of harm from their prescribed medications [27]. The current system operates in real time to detect if there is a risk of harm from prespecified conditions, for example, if methotrexate is prescribed without a coprescription for folic acid or if allopurinol is prescribed at a dose of >200 mg/day to a patient with chronic renal insufficiency. Clinicians are informed of each alert and then decide whether to take action or inform patients. Conporto has developed the capacity to contact patients directly via text and email, with patient consent. This patient contact is triggered when a patient requests a prescription from their general practice and is currently used to inform patients that their prescription is ready for collection. This function could also be used to provide patients with more information about their medications.

We have developed a printable information sheet and an online learning activity for patients. Patients, general practitioners, pharmacists, and a patient education provider have contributed to the development of these resources, which aim to inform patients about their elevated risk of harm from NSAIDs and discourage them from using over-the-counter NSAIDs. A randomized controlled trial (RCT) was proposed to examine the effect of giving at-risk patients this information directly, without needing their health care practitioners to provide it. This trial aims to assess the impact of providing an information package about avoiding anti-inflammatory medicines to patients at risk of renal damage from the “triple whammy,” in particular, the impact on anti-inflammatory knowledge and self-reported behavior. However, we have a number of concerns that we need to address before carrying out the full RCT.

First, it is unknown if our recruitment methods will be successful in enrolling a representative sample of the target population. Second, a low response rate of online surveys is a common concern. Having a better understanding of the survey response rate will help us determine the number of participants needed for the full trial. Third, while we have developed the information package with some patient input, it has not been formally evaluated by patients. Fourth, we do not know whether the survey questions are appropriate to assess the impact of the intervention. Therefore, we plan to conduct this randomized feasibility trial to assess the feasibility of the intervention and pilot the recruitment methods and the use of surveys for assessing the impact of the intervention. The results of this feasibility trial will help refine our methods prior to commencing a definitive RCT.

The primary aim of this study is to assess the feasibility of conducting an RCT. The RCT will investigate the effect of providing a patient information package about NSAIDs to patients at increased risk of renal damage because of their medications. The feasibility trial will elucidate any issues that could impair our capacity to answer the aims of the full trial.

The feasibility trial aims to (1) pilot the procedures for recruiting patients and providing patient information online to assess the number of eligible participants and the recruitment rate, assess the characteristics of participants who are enrolling in the trial, identify any technical challenges for patients assessing information online, and assess the drop-out rate in each group; (2) assess the acceptability of the patient information package to assess if patients trust and understand the information provided and think it is relevant to them; and (3) pilot the use of the survey for assessing the effects of providing patients information about the risk of NSAIDS to obtain preliminary data of the survey responses to help with sample size calculation in the full trial, assess the response rates of the surveys, and assess the suitability of survey questions to measure the impact of the intervention.

## Methods

**Figure 1:**
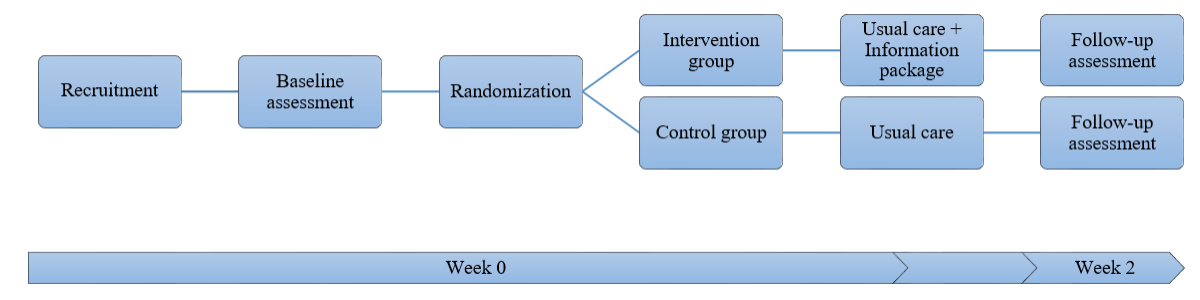
Flow chart of study protocol.

This will be a two-armed randomized feasibility trial. The trial will be conducted according to the steps outlined in Figure 1.

### Recruitment

Conporto is used by general practices across New Zealand. Patients attending general practices using Conporto in the Northland region will be eligible to participate in the randomized feasibility trial. Northland was chosen as Conporto is used widely in the region. Northland is a subtropical region in New Zealand, with a population of 179,000. Compared to the rest of New Zealand, Northland is more rural, is poorer, and has a higher proportion of people of Maori ethnicity [28]. Recruitment will be undertaken at the individual level. We aim to recruit 50 patients (25 patients in each trial arm) to pilot the use of the surveys. Participants will be allocated a unique study identification number to preserve anonymity.

Patients will be identified as having increased risk of harm from NSAIDs from their current prescriptions. Patients prescribed at least 2 of the 3 “triple whammy” medications (ie, an ACE-i or ARB plus a diuretic) will be identified by Conporto. Patients will be eligible to participate in this study if they were prescribed these medications concurrently in the past 3 months, are over 18 years old, and if they have signed up to receive Conporto’s email messages. Textbox 1 describes participant inclusion and exclusion criteria. All eligible patients will be invited to participate in the study via email from Conporto.

**Participant eligibility criteria.**

**Inclusion criteria**

- Age 18 years or older
- Angiotensin-converting enzyme inhibitor (ACE-i) or angiotensin II receptor blockers (ARB) + diuretic in the past 3 months
- Signed up to receive Conporto alerts

**Exclusion criteria**

- Fails to complete enrollment

### Randomization and Blinding

Following recruitment to the study, patients will be automatically randomized 1:1 to either the control or intervention group within each practice. Patients will not be advised to which group they are allocated. Stata software will be used to generate the randomization sequence, which will be allocated using REDCap (Research Electronic Data Capture) software. REDCap software will also be used to administer all the surveys. REDCap is a secure, web-based survey tool suitable for data requiring high-security storage (ie, patient data). Nonrespondents at each stage will be emailed 2 further invitations to participate, at 1-day intervals.

### Baseline Assessment

The study invitation email will contain a link to the baseline assessment (Multimedia Appendix 1). The first page of the assessment contains a consent form approved by the University of Otago Human Ethics Committee. Once the consent form is completed, the rest of the form opens automatically to request demographic information, information about current medications and NSAID use, a validated single-item health literacy assessment [29], a validated 4-item medicine use and self-efficacy questionnaire [23], a validated NSAID knowledge assessment [30], and a self-report of NSAID use in the preceding fortnight.

### Study Intervention

Within 24 hours of completing the baseline assessment, intervention group patients will be emailed a link to a webpage containing a fully accessible, online, interactive learning activity and a downloadable PDF [31]. Patient information about avoiding anti-inflammatories and an online, interactive learning activity have been developed by SL in conjunction with the Health Navigator editorial board, their pharmacist, and the Health Navigator patient panel in an iterative process. The webpage is hosted by Health Navigator New Zealand, a nonprofit initiative that provides curated health information overseen by the Health Navigator Charitable Trust. Both the control and intervention groups will receive usual general practice care from their own primary care team during the trial. After the trial is completed, control group patients will also be sent an email with a link to the information package, so all patients in the study eventually have access to that resource.

### Follow-Up Assessment

All patients will be sent a follow-up survey after 2 weeks that repeats 2 survey items from the baseline assessment: the validated NSAID knowledge assessment [30] and the self-report of NSAID use in the preceding fortnight

Patients in the intervention arm will also complete an additional survey to evaluate the information package. This evaluation survey is based on Normalization Process Theory (NPT) concepts (Multimedia Appendix 1) [32]. NPT has been successfully used in the assessment and implementation of multiple patient-facing complex health interventions [33-35] and to evaluate other primary care initiatives aiming to reduce kidney injury [36,37].

### Measures

We will report the number and percentage of patients who are eligible to participate in the trial and those who consent to participate. For participants in each of the study arms, we will report the response and drop-out rates for each of the surveys. The number of patients who open the information package will also be recorded via the website analytic data from the Health Navigator website [38]. The numbers of patients who interact with the education package will be reported, together with the patient evaluation of it. NSAID knowledge scores and self-reports of NSAID use will be measured at baseline and 2-week follow-up.

### Statistical Analysis

Descriptive analyses will be carried out to summarize the characteristics of the participants in each study arm. Preliminary data will be obtained for the NSAID knowledge scores and self-reported NSAID use at baseline and follow-up to guide sample size calculation for the full trial. If numbers allow, linear mixed models will be used to compare the changes in the mean score on the NSAID knowledge questionnaire and self-reported NSAID use between the 2 arms.

## Results

Funding has been obtained from the Health Research Council of New Zealand (HRC 18-031). The University of Otago Human Research Ethics Committee (H21/016) has approved this trial. Consultation has been undertaken with The Ngai Tahu research consultation committee. The trial commenced on April 12, 2021.

The expected outcomes include that we will determine the uptake, acceptability, and self-reported effects of providing patients with information about the risk of NSAIDs via a webpage; determine the feasibility of conducting this research via an online survey, and these data will be used to apply for further research funding; and publications and conference presentations about the trial results.

## Discussion

This feasibility trial will help us determine whether it is practical to conduct a nationwide RCT. It will help refine the information package. Additionally, it may provide preliminary data to help understand whether providing information directly to patients increases their knowledge or changes their behavior.

### Strengths and Limitations

This trial will evaluate the entire implementation process, testing trial processes and the acceptability of the intervention. The results of this feasibility trial will ensure best use of limited health research funding for any future similar studies. Publication of this work will help other researchers considering conducting similar patient-focused research.

Language, education, and technology can be barriers to health literacy [39]. The main limitation of this research is that patients with the greatest barriers to health literacy are likely to experience those same barriers in accessing the proposed intervention and participating in this research project. The research project and proposed intervention were developed only in English due to financial constraints. If this intervention is successful in English on full testing, it is hoped funding will be made available to translate the intervention into different languages (eg, Maori, Samoan, and Tongan in the first instance).

A potential risk of this work is that patients experience anxiety or distress when they realize they are at increased risk of harm from medication. This risk is mitigated by the ubiquitous nature of health information readily available to patients and the repeated encouragement for participants to discuss their concerns with their health care providers.

### Comparison With Prior Work

Only a handful of feasibility trial protocols have been published to date in the field of primary care medication safety. Feasibility studies focus on testing and evaluating the study processes to assess “can it work?”, while pilot studies focus on outcomes to assess the effectiveness of the intervention, or “does the intervention show promise?” [40]. Feasibility studies are particularly important for complex interventions, which risk being undermined by problems that could be otherwise sorted at an exploratory stage, such as the target population failing to engage with the study or intervention [41]. Testing the feasibility of a project improves the chance of success of a larger trial, thus ensuring better use of funding and reducing the risk of harms arising from the intervention or study processes [41].

### Conclusions

This feasibility trial protocol describes our plan to test trial processes prior to commencing a nationwide RCT. It will provide important information about the acceptability of the patient information package. We anticipate this work will offer a useful model for other researchers attempting similar work.

## Appendix

Multimedia Appendix 1 Survey questions.

## Abbreviations

ACE-i: angiotensin-converting enzyme inhibitors
ARB: angiotensin II receptor blockers
NPT: Normalization Process Theory
NSAID: non-steroidal anti-inflammatory drug
RCT: randomized controlled trial
REDCap: Research Electronic Data Capture

## References

[1] Zhang X, Donnan PT, Bell S, Guthrie B (2017). Non-steroidal anti-inflammatory drug induced acute kidney injury in the community dwelling general population and people with chronic kidney disease: systematic review and meta-analysis. BMC Nephrol.

[2] Lapi F, Azoulay L, Yin H, Nessim SJ, Suissa S (2013). Concurrent use of diuretics, angiotensin converting enzyme inhibitors, and angiotensin receptor blockers with non-steroidal anti-inflammatory drugs and risk of acute kidney injury: nested case-control study. BMJ.

[3] (2019). Polypharmacy in poeple aged 65 and over. Health Quality & Safety Commission New Zealand.

[4] (2017). Avoiding the triple whammy in primary care: ACE inhibitor/ARB + diuretic + NSAID. bpacnz.

[5] Mullan J, Weston KM, Bonney A, Burns P, Mullan J, Rudd R (2017). Consumer knowledge about over-the-counter NSAIDs: they don't know what they don't know. Aust N Z J Public Health.

[6] Hughes L, Whittlesea C, Luscombe D (2002). Patients' knowledge and perceptions of the side-effects of OTC medication. J Clin Pharm Ther.

[7] Guirguis K (2010). The use of nonprescription medicines among elderly patients with chronic illness and their need for pharmacist interventions. Consult Pharm.

[8] Young A, Tordoff J, Smith A (2017). 'What do patients want?' Tailoring medicines information to meet patients' needs. Res Social Adm Pharm.

[9] Dickinson R, Hamrosi K, Knapp P, Aslani P, Sowter J, Krass I, Raynor DK (2013). Suits you? A qualitative study exploring preferences regarding the tailoring of consumer medicines information. Int J Pharm Pract.

[10] Lowe DB, Sharma AK, Leathley MJ (2007). The CareFile Project: a feasibility study to examine the effects of an individualised information booklet on patients after stroke. Age Ageing.

[11] Kusch MK, Haefeli WE, Seidling HM (2018). How to meet patients' individual needs for drug information - a scoping review. Patient Prefer Adherence.

[12] Young A, Tordoff J, Leitch S, Smith A (2018). Do health professionals tell patients what they want to know about their medicines?. Health Education Journal.

[13] Leitch S, Smith A, Crengle S, Stokes T (2021). The views of New Zealand general practitioners and patients on a proposed risk assessment and communication tool: a qualitative study using Normalisation Process Theory. Implement Sci Commun.

[14] Brounéus F, Macleod G, Maclennan K, Parkin L, Paul C (2012). Drug safety awareness in New Zealand: public knowledge and preferred sources for information. J Prim Health Care.

[15] Young A, Tordoff J, Moore S, Smith A (2019). Patients’ views of general practitioners’ provision of medicine information leaflets. J Prim Health Care.

[16] Franken M, Hunter J (2013). The construction of participants, causes and responses in ‘problematic’ health literacy situations. JALPP.

[17] Edwards A, Matthews E, Pill R, Bloor M (1998). Communication about risk: diversity among primary care professionals. Fam Pract.

[18] Phueanpinit P, Pongwecharak J, Sumanont S, Krska J, Jarernsiripornkul N (2017). Physicians' communication of risks from non-steroidal anti-inflammatory drugs and attitude towards providing adverse drug reaction information to patients. J Eval Clin Pract.

[19] (2010). Korero Marama: Health Literacy and Maori. Results from the 2006 Adult Literacy and Life Skills Survey. Ministry of Health.

[20] Berkman ND, Sheridan SL, Donahue KE, Halpern DJ, Crotty K (2011). Low health literacy and health outcomes: an updated systematic review. Ann Intern Med.

[21] Stacey D, Légaré F, Lewis K, Barry MJ, Bennett CL, Eden KB, Holmes-Rovner M, Llewellyn-Thomas H, Lyddiatt A, Thomson R, Trevena L (2017). Decision aids for people facing health treatment or screening decisions. Cochrane Database Syst Rev.

[22] Edwards AGK, Naik G, Ahmed H, Elwyn GJ, Pickles T, Hood K, Playle R (2013). Personalised risk communication for informed decision making about taking screening tests. Cochrane Database Syst Rev.

[23] Cameron KA, Ross EL, Clayman ML, Bergeron AR, Federman AD, Bailey SC, Davis TC, Wolf MS (2010). Measuring patients' self-efficacy in understanding and using prescription medication. Patient Educ Couns.

[24] Lamarche L, Tejpal A, Mangin D (2018). Self-efficacy for medication management: a systematic review of instruments. Patient Prefer Adherence.

[25] Wilson EAH, Makoul G, Bojarski EA, Bailey SC, Waite KR, Rapp DN, Baker DW, Wolf MS (2012). Comparative analysis of print and multimedia health materials: a review of the literature. Patient Educ Couns.

[26] Chapman E, Haby MM, Toma TS, de Bortoli MC, Illanes E, Oliveros MJ, Barreto JOM (2020). Knowledge translation strategies for dissemination with a focus on healthcare recipients: an overview of systematic reviews. Implement Sci.

[27] Conporto H (2018). Conporto EDM (Event Detection & Mitigation) Proof of Concept: Final Report. Patients First.

[28] Stats NZ (2018). Northland Region. New Zealand Government.

[29] Morris NS, MacLean CD, Chew LD, Littenberg B (2006). The Single Item Literacy Screener: evaluation of a brief instrument to identify limited reading ability. BMC Fam Pract.

[30] Jang SM, Jiang R, Grabe D, Pai AB (2019). Assessment of literacy and numeracy skills related to non-steroidal anti-inflammatory drug labels. SAGE Open Med.

[31] Leitch S (2021). Anti-inflammatories learning activity. Health Navigator New Zealand.

[32] Murray E, Treweek S, Pope C, MacFarlane A, Ballini L, Dowrick C, Finch T, Kennedy A, Mair F, O'Donnell C, Ong BN, Rapley T, Rogers A, May C (2010). Normalisation process theory: a framework for developing, evaluating and implementing complex interventions. BMC Med.

[33] May CR, Mair FS, Dowrick CF, Finch TL (2007). Process evaluation for complex interventions in primary care: understanding trials using the normalization process model. BMC Fam Pract.

[34] May CR, Cummings A, Girling M, Bracher M, Mair FS, May CM, Murray E, Myall M, Rapley T, Finch T (2018). Using Normalization Process Theory in feasibility studies and process evaluations of complex healthcare interventions: a systematic review. Implement Sci.

[35] McEvoy R, Ballini L, Maltoni S, O'Donnell CA, Mair FS, Macfarlane A (2014). A qualitative systematic review of studies using the normalization process theory to research implementation processes. Implement Sci.

[36] Martindale A, Elvey R, Howard SJ, McCorkindale S, Sinha S, Blakeman T (2017). Understanding the implementation of 'sick day guidance' to prevent acute kidney injury across a primary care setting in England: a qualitative evaluation. BMJ Open.

[37] Morris RL, Ashcroft D, Phipps D, Bower P, O'Donoghue D, Roderick P, Harding S, Lewington A, Blakeman T (2016). Preventing Acute Kidney Injury: a qualitative study exploring 'sick day rules' implementation in primary care. BMC Fam Pract.

[38] Health Navigator New Zealand.

[39] Hunter J, Franken M (2012). Health Literacy as a Complex Practice. LNS.

[40] Orsmond GI, Cohn ES (2015). The Distinctive Features of a Feasibility Study: Objectives and Guiding Questions. OTJR (Thorofare N J).

[41] Hallingberg B, Turley R, Segrott J, Wight D, Craig P, Moore L, Murphy S, Robling M, Simpson SA, Moore G (2018). Exploratory studies to decide whether and how to proceed with full-scale evaluations of public health interventions: a systematic review of guidance. Pilot Feasibility Stud.

