# Supplementary material for: Using an Information Package to Reduce Patients’ Risk of Renal Damage: Protocol for a Randomized Feasibility Trial
Source: JMIR Res Protoc. 2021 Apr 30;10(4):e29161. doi: 10.2196/29161 (PMC8122288; doi:10.2196/29161)

# Appendix 1

## Baseline survey


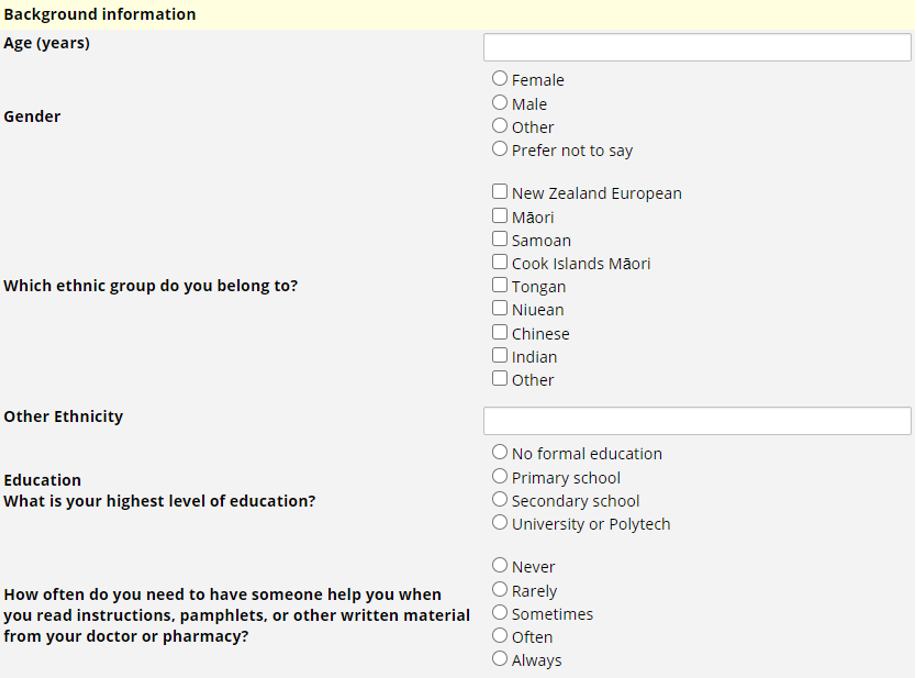


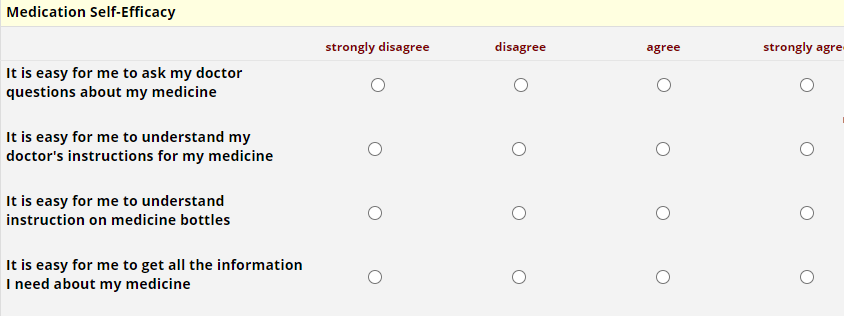


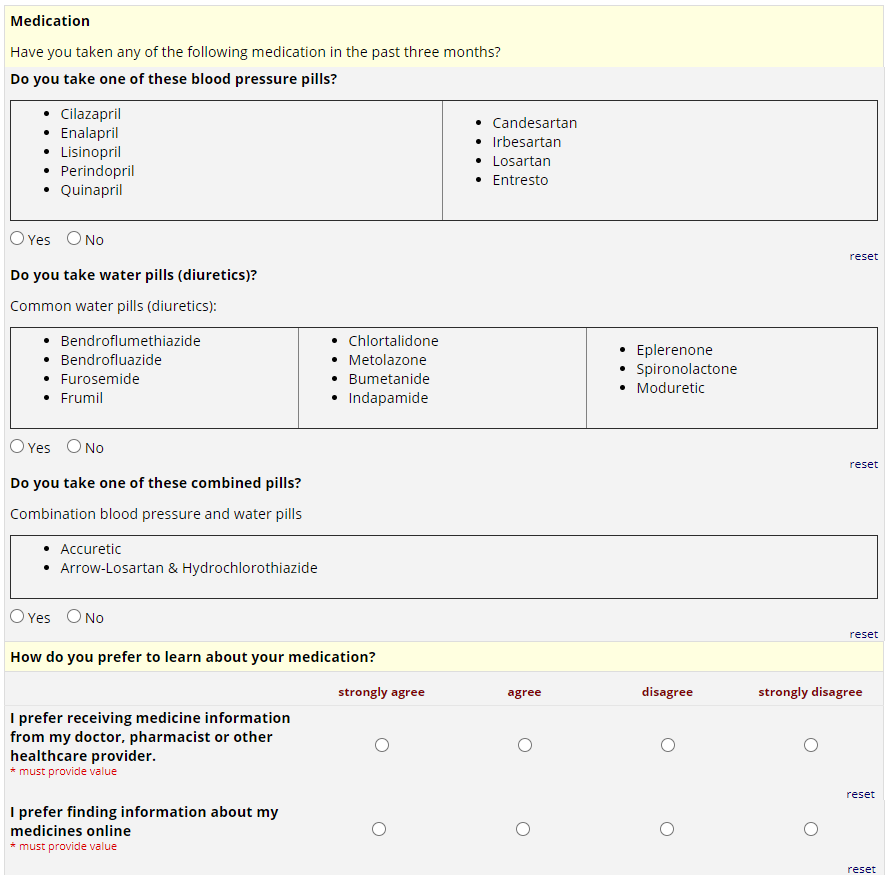


## The following items are completed in both the baseline and follow-up surveys:


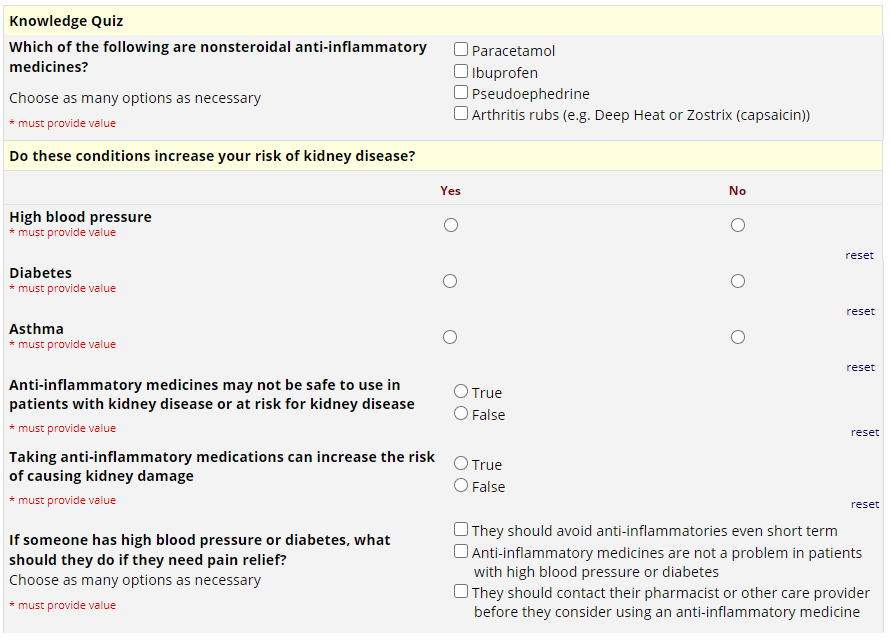


Self reported action
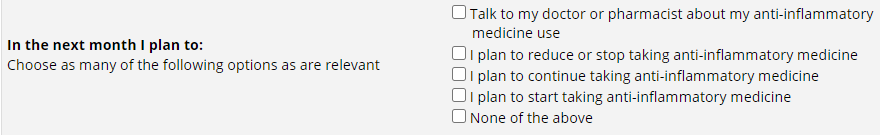


## Anti-inflammatory learning package evaluation

##
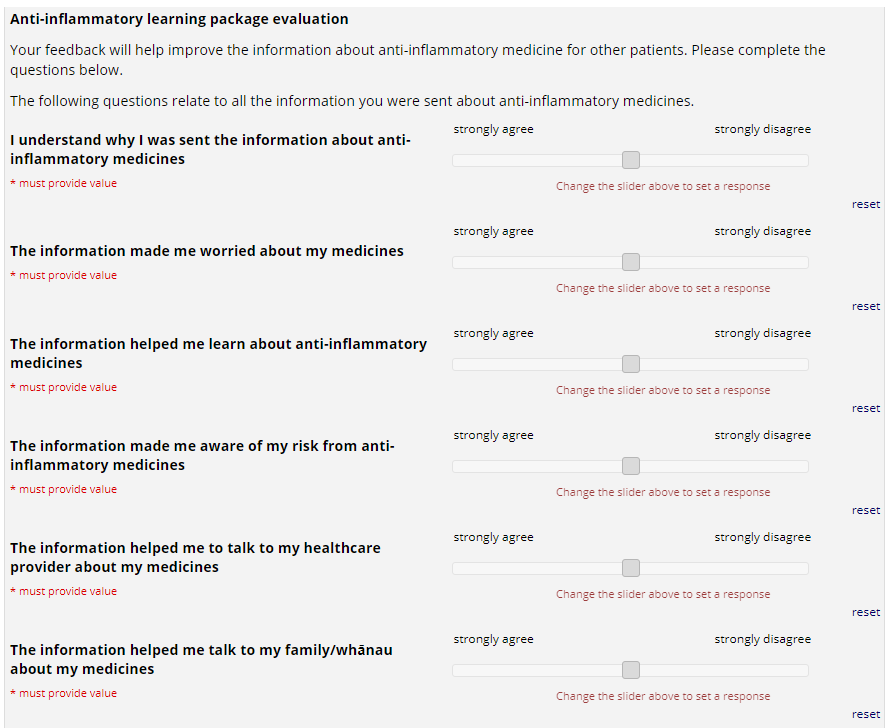

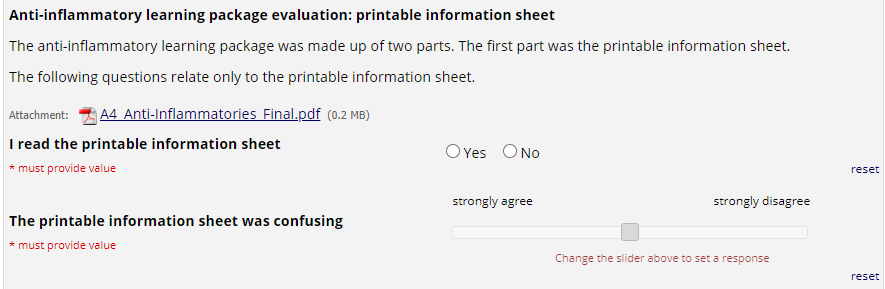


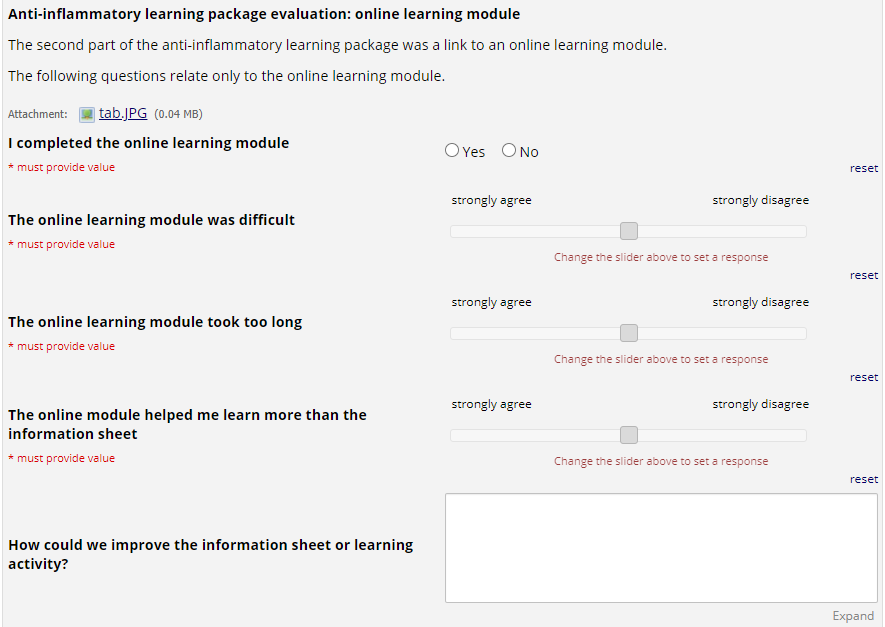

Supplement: Multimedia Appendix 1 [file resprot_v10i4e29161_app1.docx]
